# Analysis of Comprehensive Edible Quality and Volatile Components in Different Varieties of Cooked Highland Barley

**DOI:** 10.3390/foods14101690

**Published:** 2025-05-10

**Authors:** Caijiao Li, Jun Li, Wengang Zhang, Bin Dang, Xijuan Yang

**Affiliations:** 1 Academy of Agriculture and Forestry Sciences, Qinghai University, Xining 810016, China; 2019990054@qhu.edu.cn (C.L.); 2017990098@qhu.edu.cn (W.Z.); 2008990019@qhu.edu.cn (B.D.); 2 Key Laboratory of Qinghai Province Tibetan Plateau Agric-Product Processing, Qinghai University, Xining 810016, China; 3 College of Agriculture and Animal Husbandry, Qinghai University, Xining 810016, China; 4 Xining No. 1 Middle School, Xining 810000, China

**Keywords:** highland barley, comprehensive edible quality, GC-IMS, volatile flavor

## Abstract

Twenty-two types of highland barley (HB) raw materials (including 10 common varieties and 5 main planting regions in the Qinghai province) were selected as the experimental materials to investigate their differences in the cooking characteristics, sensory quality, and characteristic flavor of cooked HB. The key volatile flavor components were identified using Gas Chromatography–Ion Mobility Spectroscopy (GC-IMS) combined with relative odor activity value (ROAV) analysis. The results indicated that the highland barley raw materials of Kunlun 15 (M5), Kunlun 14 (M9), Chaiqing 1 (M13) and Kunlun 14 (M14), and Chaiqing 1 (M20) and Kunlun 15 (M21) showed superior cooking quality, texture, and sensory scores. A total of 44 volatile flavor compounds were identified, including 16 aldehydes, 10 alcohols, 9 ketones, 7 esters, 1 acid, and 1 furan. Among these, 13 aldehydes, 4 alcohols, 4 ketones, 7 esters, and 1 furan were found across different cooked HB samples. Notably, ethyl, ethyl 2-methylbutanoate dimer, 2-methylbutanoic acid methyl ester, 2-butanone, 1-octen-3-ol, 1-pentanol dimer, and 2-pentyl furan contributed more significantly to the overall volatile profile. Cluster analysis combining principal component analysis revealed that Kunlun 16 (M16), Kunlun 17 (M17), Kunlun 14 (M18), Kunlun 15 (M19), as well as Chaiqing 1 (M20) and Kunlun 15 (M21), were the most suitable raw materials for cooking due to their better cooking quality, sensory attributes, and flavors, followed by Kunlun 15 (M10) and Kunlun 18 (M12), and Chaiqing 1 (M13) and Kunlun 14 (M14). These findings could help us identify specific HB varieties in corresponding regions with advantages, thus providing a theoretical basis for cooking HB.

## 1. Introduction

Highland barley (*Hordeum vulgare* L. var. *nudum* Hook. f.), also known as naked barley due to its easily separated inner and outer glume shells, is a cereal crop native to western China, particularly the Tibetan Plateau region [1,2,3]. It is a distinctive wholegrain valued for its exceptional nutritional and health-promoting properties, characterized by the “three highs and two lows” (high protein, high dietary fiber, high vitamin, low fat, and low polysaccharide). Compared with other *Triticeae* crops, it is also rich in functional components such as phenols, β-glucan, xylan, gamma-aminobutyric acid (GABA), plant sterols, tocopherols, and anthocyanins [4,5,6], which contributes to its popularity as a valuable food crop.

Cooked highland barley (HB) is a product that undergoes a series of processes, such as cleaning, peeling, grading, and color selection, which is one of the main products for HB to be transported outside the region of the plateau [7]. Cooking is a common step before consuming HB. After cooking, HB develops a unique flavor, which is related to the abundant volatile compounds produced during the processing due to changes in nutrients. However, the comprehensive edible quality and flavor compounds in HB are complex and are easily influenced by the types of raw materials and pre-processing methods [8]. Analyzing the comprehensive edible quality and volatile flavors in different types of cooked HB is crucial for understanding the formation of its characteristic flavor and guiding the regulation of HB quality. Previous studies have reported the influence of processing on the volatile flavor compounds of highland barley [8,9]. Zhang et al., examined the effect of milling degree on the nutritional quality and sensory quality and volatile flavor compounds of HB and concluded that an increase in milling degree led to a decrease in protein, fat, fiber, ash, total phenol, and total flavonoid contents, and that the cooked HB achieved its optimal overall sensory quality when the milling mass loss rate reached 14.54%. In addition, 35 volatile flavor substances were identified, including aldehydes, ketones, alcohols, and esters [10]. Lee et al. studied the cooking characteristics and sensory evaluation of the germinated barley, revealing that the hardness of cooked barley tended to decrease after the germination of barley and that barley that germinated for 2–4 days was the most suitable (overall acceptance) for cooking rice based on the sensory evaluation [11]. In another study on the effect of pearling on the nutritious composition and cooking quality of highland barley conducted by Li et al. [12], it was shown that pearling could significantly shorten the cooking time of highland barley and the appropriate degree of pearling (DOP) could improve the cooking and sensory quality of highland barley and better retain its nutrients. Due to the complexity of the climate in the Qinghai Tibet Plateau region and the diversity of highland barley germplasm resources, current research on the suitability of HB processing is still not comprehensive enough. Especially limited research has been conducted on the impact of the differences in highland barley varieties produced in different main cultivation areas on the comprehensive edible quality and volatile flavor components of HB, which hinders the high-quality development of HB products in the Qinghai region.

22 major highland barley raw materials (including 10 common varieties and 5 main planting regions in the Qinghai province) were selected underthe interaction background of varieties and objective planting conditions to evaluate their differences in the cooking characteristics, sensory quality, and characteristic flavor in this study. Gas Chromatography–Ion Mobility Spectroscopy (GC-IMS), combined with hierarchical cluster analysis (HCA), principal component analysis (PCA), and relative odor activity value (ROAV), was employed to analyze the key volatile flavor compounds of HB and the variations associated with different samples. Based on the findings regarding comprehensive edible quality and key volatile flavors, the most suitable highland barley materials (specific varieties in specific areas) forcooking HB were identified, providing theoretical support and guidance for the scientific processing of HB.

## 2. Materials and Methods

### 2.1. Samples

Twenty-two highland barley raw materials offered by the Qinghai Academy of Agriculture and Forestry Sciences in 2024 from different regions of Qinghai (cultivated in Xining City, Dulan County of Haixi Autonomous Prefecture, Guinan County and Guide County of Hainan Prefecture, and Menyuan County of Haibei Prefecture) were selected because they represented the main cultivated highland barley types in Qinghai region. They are rich in nutrients, possessing certain market development potential for the development of highland barley industry in Qinghai region [13]. Detailed information on the varieties and planting areas is provided in Table 1. For the convenience of expression, all 22 highland barley raw materials were numbered as different independent varieties.

### 2.2. Preparation of the Cooked HB

The preparation of the cooked HB followed the procedure outlined by Li et al. [12] with a slight modification. Based on our previous research [10], the grains were ground and peeled to achieve a peeling rate of 15 ± 0.30%, when the nutritional function and flavor characteristics of cooked HB were relatively the best. The HB was washed thoroughly and soaked in water at 1:10 (g/mL) for 2 h. Then, 40 g soaked grains was added to 800 mL boiling water (98 ± 2 °C) for 30 min. After cooking, the HB was drained, cooled to room temperature, and subjected to sensory evaluation.

### 2.3. Determination of Cooking Quality

The water absorption and volume expansion rates of HB were measured according to the method proposed by Zhang et al. [14] with a slight modification. The HB washed thoroughly was soaked in water at 1:10 (g/mL) for 2 h. Then, approximately 10 g highland barley samples (recorded as M_1_) were accurately weighed and added to 200 mL boiling water (98 ± 2 °C) for 30 min. After cooling, the cooked HB was weighed (recorded as M_2_). The volume of uncooked and cooked HB was determined using the volume replacement method (recorded as V_1_ and V_2_, respectively). The following formulae were used:

(1) $$Water\;absorption\;rate=\frac{M_{2}-M_{1}}{M_{1}}\times 100\%$$

(2) $$Volume\;expansion\;rate=\frac{V_{2}-V_{1}}{V_{1}}\times 100\%$$

### 2.4. Determination of Texture Characteristics

Texture characteristics were determined based on the methods described by Liu et al. [15] with slight modifications. Four cooked HBgrains were placed in a radial pattern on the test bench, the texture characteristics including hardness, adhesiveness, cohesiveness, elasticity, stickiness, and chewiness were measured using a texture analyzer (FTC TMS-PRO, Food Technology Corporation, Sterling, VA, USA). The texture profile analysis (TPA) procedure used a 25 mm probe height, 0.5 N starting force, 1 mm/s detection speed, and 40% deformation component. The hardness was the absolute peak load recorded during the compression cycle, adhesiveness was total energy invested to completely break contact between the test probe and the sample during decompression cycle, cohesiveness was the ratio of positive force during the second compression cycle to that of the first compression cycle, and elasticity was the displacement between negative crossover on decompression cycle to adhesive force. Stickiness and chewiness were calculated following formulae below:

(3) Stickiness=hardness×cohesiveness

(4) Chewiness=hardness×cohesiveness×elasticity

### 2.5. Sensory Evaluation

The sensory evaluation of the cooked HB was performed by a panel consisting of 10 members (ages 18 to 28), all of whom majored in food concerned speciality [16]. They all gained extensive experience in sensory characterization of various food samples. Prior to the analysis, every evaluator attended eight training sessions (spending 2 h each) during the two weeks until they had all gained sufficient experience in sensory analysis and were relatively familiar with sensory evaluation of the cooked HB. Evaluations were conducted individually in temperature-controlled compartments. The sensory evaluation of the cooked HB was conducted in accordance with the Chinese national standard (GB/T 15682-2008) [17] and the method conducted by Gao et al. [18] with slight modifaictions. Five aspects including smell, color, appearance structure, palatability, and taste were assessed. The definitions and assessment criteria for each indictor are detailed in Table 2, every evaluator scored the sample three times based on that and the average score was the comprehensive score of each evaluator. The final sensory score of the cooked HB was calculated according to the comprehensive rating results of each evaluator. Individual evaluators with larger evaluation errors (different from the average by more than 10 points) would be discarded and then the average value would be recalculated.

### 2.6. GC-IMS Analysis of Volatile Flavor Compounds

A gas chromatography–ion mobility spectrometer (FlavourSpec^®^) manufactured by G.A.S. (Berlin, Germany) was employed for the analysis of volatile flavor compounds. The chromatographic column used in this study was WAX (30 m × 0.53 mm, 1 μm) provided by RESTEK Company (Bellefonte, PA, USA). The GC-IMS analysis of volatile flavor compounds was performed following the method outlined by Zhang et al. [10]. Approximately 1.0 g of HB, soaked for 2 h, was placed in a 20 mL headspace vial and incubated for 30 min at 100 °C, then 500 μL sample was automatically injected with an injection needle temperature of 95 °C and an incubation speed of 500 rpm.

The detailed experimental parameters were set as follows: the column temperature was set at 60 °C and N_2_ with purity higher than 99.999% was used as the carrier gas. The carrier gas flow started with an initial 2 min flow rate of 2.0 mL/min, followed by a linear increase to 10.0 mL/min in the next 8 min, and finally reached 100.0 mL/min over the next 10–20 min with a constant drift gas flow rate of 150 mL/min and IMS temperature of 45 °C.

Data analysis of GC-IMS was conducted using the laboratory analytical software equipped to the instrument including VOCal and three plugins: Reporter, Gallery Plot, and Principal Component Analysis (PCA) [19]. This enabled the construction of three-dimensional, two-dimensional, and fingerprint difference spectra. Each point in the analyzed spectra represented a specific volatile flavor. Volatile compounds were identified by comparing retention index and the drift time (DT, which is the time it takes for ions to reach the collector through the drift tube, in milliseconds) with the NIST and IMS databases incorporated in the VOCal software (NIST2014). The relative content of each volatile flavor was calculated by a ratio of the peak volume of each volatile flavor to the total volume of peaks in GC-IMS chromatograms.

### 2.7. Evaluation of Key Volatile Flavor Compounds

It is hard to determine the “key flavor” compound based on relative concentrations of volatile compounds because many important aroma-active compounds remain under the threshold or under the detection limit. Some compounds may be in high concentration but make less contribution to overall flavor. Therefore, the contribution of different volatile flavors to the overall flavor of the samples was evaluated using the relative odor activity value (ROAV) method based on the relative concentrations of volatile compounds determined by GC-IMS analysis. The larger the ROAV, the greater the contribution of the compound to the overall flavor of the sample, with a maximum ROAV value of 100 [20]. Herein, all volatile components in the sample had ROAVs ≤ 100, and the components with ROAV ≥ 1 were considered to be the key flavor compounds, of which greater than 0.1 and smaller than 1 had a slight effect on the overall flavor of the sample, and smaller than 0.1 were potential flavor compounds [21]. The ROAV of other compounds was calculated using the following formula:

(5) $${ROAV}_{i}\approx 100\times C_{i}/C_{s}\times T_{s}/T_{i}$$

where C_i_ and T_i_ is the relative content of component i (%) and the sensory threshold of component i (μg/kg), respectively; C_s_ represents the relative content which contributes most to the main flavor of the sample (%); and T_s_ is the sensory threshold which contributes most to the main flavor of the sample (μg/kg).

### 2.8. Data Processing

Experimental data in this study were expressed as the mean ± standard deviation (SD) from three replicate samples (n = 3). Significant differences were determined using Duncan’s test (*p* < 0.05). Hierarchical cluster analysis and principal component analysis were performed using Origin 2021. The analysis of variance (ANOVA) of variety (V), environment (E) and V × E interaction on the nutritional quality of HB was made by IBM SPSS Statistics 23.0. Statistical analyses and chart plotting were statistically conducted by IBM SPSS Statistics 23.0 and Origin 2021.

## 3. Results and Discussion

### 3.1. Analysis of HB Cooking Quality

**Figure 1:**
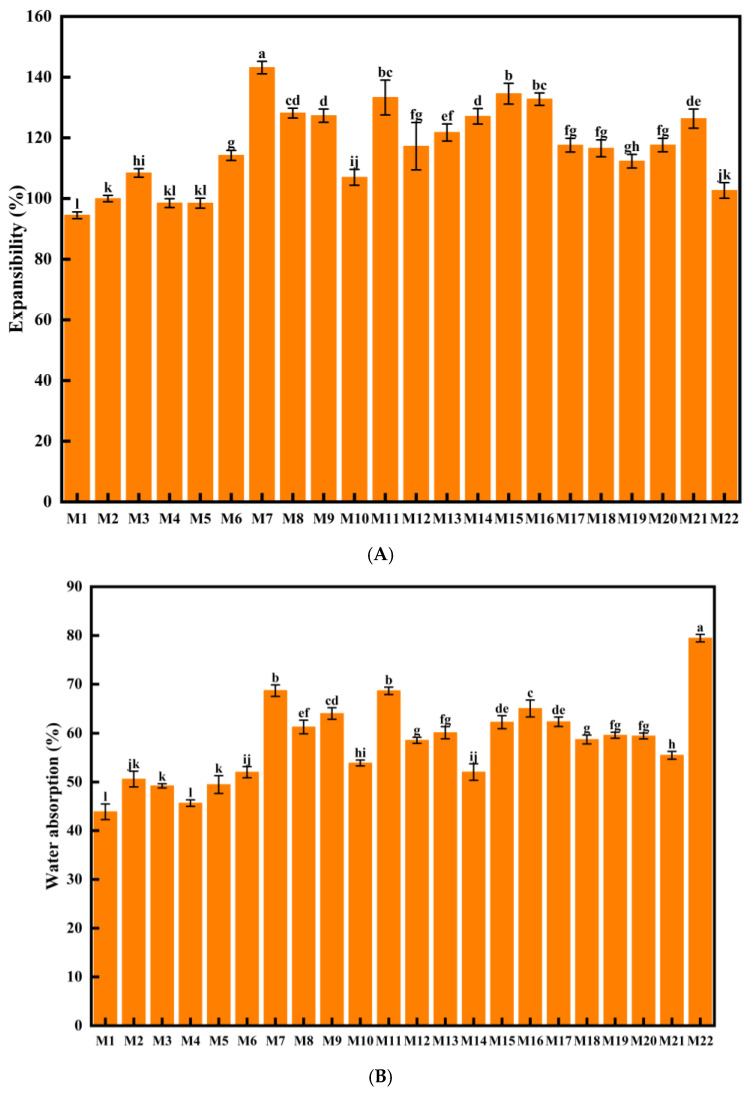
The expansibility (**A**) and water absorption (**B**) of cooked HB. All data were expressed by mean standard deviation. Different lowercase letters indicate statistical differences between samples at *p* < 0.05 level.

The water absorption rate indicates the water absorption capacity of HB during the cooking gelatinization process, which is an important indicator to evaluate the cooking conditions. It is primarily influenced by factors such as grain freshness, amylose content, protein content, and moisture level [22]. On the other hand, the volume expansion rate measures how much the HB increases in volume during cooking, which correlates with the meal yield. A higher volume expansion rate indicates a higher meal yield. As shown in Figure 1A, the average volume expansion rate of HB was approximately 120%, consistent with the findings of Lee et al. [11]. The average water absorption rate (Figure 1B) of HB was about 60%, lower than that of rice reported by Zhang et al. (200%) [14]. Significant differences in water absorption and volume expansion rates were observed among different HB varieties from the same region (*p* < 0.05). Previous studies have shown that higher levels of amylose content exhibited higher water absorption capacity but lower volume expansion rate. In addition, it was also reported that the cooking quality was related to the cultivar, soaking temperature, soaking time, the ratio of length and width of the grains, branching structure of amylopectin, as well as the specific nutritional composition and grain density of highland barley [11,14,23]. Among the varieties, Dulihuang (M7), Beiqing 8 (M8), Kunlun 14 (M9), Kunlun 15 (M10), Kunlun 17 (M11), and Kunlun 18 (M12) from Menyuan, Chaiqing 1 (M13), Kunlun 15 (M15), Kunlun 16 (M16), and Kunlun 17 (M17) from Guinan, Kunlun 14 (M18) from Guide, and Chaiqing 1 (M20) from Dulan had relatively higher water absorption and volume expansion rates than the others, mainly due to their relatively high content of amylopectin, which made them more suitable for cooking. In addition, significant differences in these rates were found between the same varieties grown in different regions (*p* < 0.05), likely due to variations in climatic conditions [24]. The basic nutritional composition of HB has a decisive impact on the quality of its processed products. ANOVA was conducted on the relevant data to further reveal the influence of variety and environment on the nutritional quality of cooked HB, and the results are shown in Table A1. It could be found that V, E, and V × E interaction all had an extremely significant impact on the grain quality and nutritional quality of highland barley, especially E played a crucial role in the formation of the basic nutritional quality of HB. The HB grown in Guinan showed the highest average volume expansion rate (126.73%), while the HB from Menyuan had a higher average water absorption rate (62.52%), which further illustrated the considerable impact of the environment on the quality of HB. Therefore, highland barley from these regions is more suitable for cooking. Notably, the only waxy highland barley, 21Y-59 (M22) from Xining, exhibited the highest water absorption rate (79.44%); it is known that waxy barley cultivars gelatinize well and there is a higher expansion rate because of its higher amylopectin content (Table A1) [11].

### 3.2. Analysis of Cooked HB Texture Characteristics

The results for the adhesion and cohesion of the different cooked HB varieties are shown in Table 3. Except for the only waxy highland barley, 21Y-59 (M22) from Xining, no significant differences in adhesion and cohesion were observed, with average values of 0.007 and 0.66, respectively, indicating that the structure of cooked HB is compact and resistant to sticking. However, significant differences (*p* < 0.05) were found in hardness, elasticity, stickiness, and chewiness among the different cooked HB varieties. In general, apparent amylose content was positively related to the hardness but negatively associated with the stickiness of the cooked HB, whereas the higher the protein content, the harder the cooked HB [25,26]. Additionally, fat, particularly polar lipids, which can serve as a bridge to join proteins and starch granules, can affect the physicochemical characteristics of cooked HB. Furthermore, other factors including variety, planting region, amylose content, and moisture content of highland barley, as well as the factors mentioned above, will have an comprehensive influence on the texture properties of HB [27,28]. It was worth noting that HB cooked from the waxy highland barley, 21Y-59 (M22), exhibited the highest hardness, elasticity, stickiness, and chewiness, showing significant differences from the other varieties, largely due to the higher amylopectin content in waxy barley compared with normal barley (Table A1). This could contribute to the higher water absorption rate, greater content leaching, and increased expansion during cooking and finally results in the higher viscosity, elasticity, and chewiness of waxy HB [29,30,31], consistent with the findings in Figure 1.

### 3.3. Sensory Evaluation of Cooked HB

**Figure 2:**
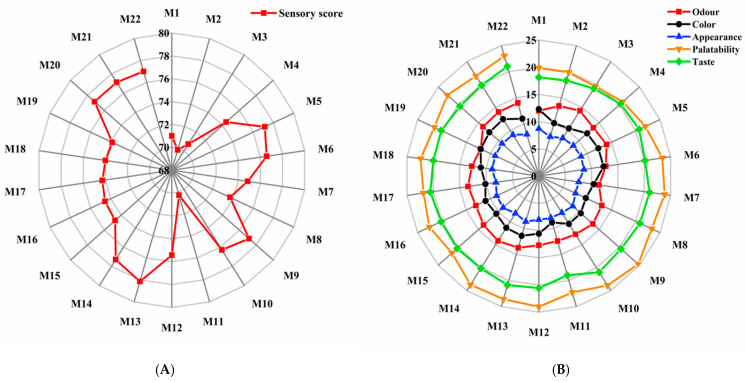
The sensory score (**A**) and sensory evaluation (**B**) of 22 cooked HB.

Generally, HB exhibited a smooth, bright surface after cooking, as shown in Figure 2, the sensory scores of cooked HB ranged from 69.86 to 78.14, with only slight variations in the coefficient of different index scores (smell, color, appearance structure, palatability, and taste) and the total score, suggesting minor distinctions in the sensory quality among different varieties of cooked HB. Varieties such as Chaiqing 1 (M1), Beiqing 9 (M2), and Dulihuang (M3) from Xining, and Kunlun 17 (M11) from Menyuan received relatively lower sensory scores due to a relatively dark and uneven color distribution, as well as their poor chewiness. In contrast, HB produced from Kunlun 15 (M5), 21Y-59 (M22), and Kunlun 18 (M6) from Xining, Kunlun 14 (M9) and Kunlun 15 (M10) from Menyuan, Chaiqing 1 (M13) and Kunlun 14 (M14) from Guinan, and Chaiqing 1 (M20) and Kunlun 15 (M21) from Dulan all scored above 75 points since they had a rich aroma, uniform and shiny color, complete grain structure, or good chewiness. The above results indicated that the sensory quality of HB was not only affected by the variety, but also significantly influenced by the cultivation area. It could also be found from the result of ANOVA that V × E interaction had an extremely significant impact on the grain quality and nutritional quality of highland barley. Therefore, cultivating specific highland barley varieties in particular regions should be considered when cultivating crops for specific processing purposes. Notably, HB from Guinan’s Chaiqing 1 (M13) achieved the highest sensory score of 78.14, indicating the best overall sensory quality, which might be related to its higher starch and protein content, as well as its tight grain state (Table A1).

### 3.4. GC-IMS Fingerprint Analysis of Cooked HB

**Figure 3:**
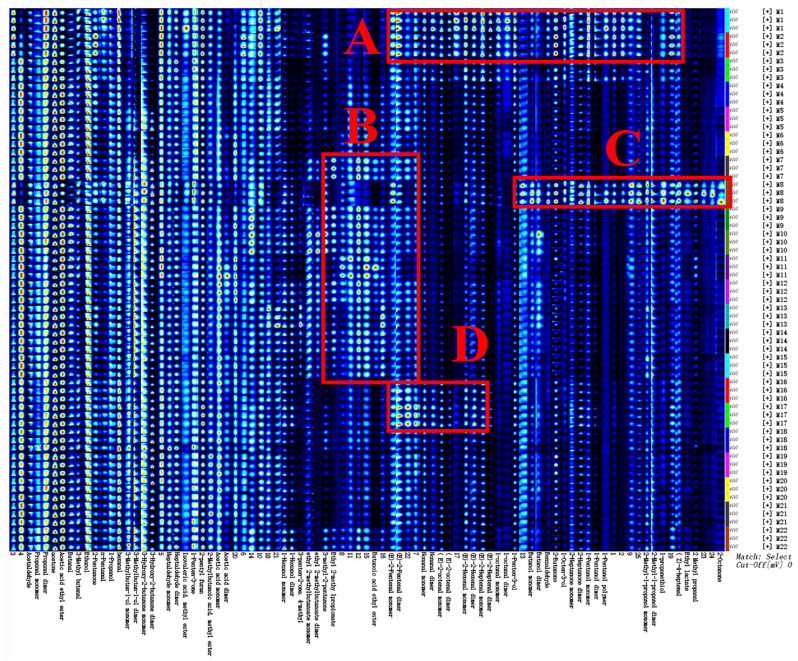
Fingerprint of volatile flavors in cooked HB. The (**A**–**D**) represent the key flavor compounds in 22 different cooked HB. Note: the flavor substances from left to right (without distinguishing monomers and dimers) were successively acetaldehyde, propanaldehyde, acetone, ethyl acetate, butyraldehyde, 3-methylbutyraldehyde, ethanol, 2-pentanone, pentalaldehyde, propanol, hexaldehyde, 3-methylbutanol, ethylacetoin, heptanaldehyde, methyl isovalerate, 1-pentene-3-one, 2-amylfuran, 2-methylbutyralate, acetic acid, hexanol, 4-methyl-3-pentene-2-ketone, ethyl 2-methylbutyrate, 3-methyl-2-pentenone, ethyl 2-methylpropionate, ethyl butyrate, e-2-pentenal, nonaldehyde, e-2-octenal, e-2-hexenal, e-2-heptenal, octanal, 1-pentene-3-ol, butanol, benzaldehyde, 2-butanone, 1-octene-3-ol, 2-heptanone, amyl alcohol, 2-methyl-1-propanol, propyl mercaptan, Z-4-heptal, ethyl lactate, 2-methylpropanal, and 2-octanone.

The volatile flavor compounds in 22 varieties of cooked HB, as identified by GC-IMS, are shown in Figure 3. A total of 87 volatile compounds were detected, of which 44 were accurately identified, including 16 aldehydes, 10 alcohols, 9 ketones, 7 esters, 1 acid, and 1 furan. Related studies have shown that, after steaming and cooking, the alcohol and aldehyde substances in cooked rice increased, which was mainly due to the boiling state during steaming and cooking, leading to the non-enzymatic browning and Maillard reaction of components [32]. These findings were also generally consistent with those reported by Zhang et al. [10], who investigated volatile flavor compounds in the cooked HB at different milling degrees.

Aldehydes are one of the main products of the Maillard reaction and fat oxidation [33], typically containing 5 to 9 carbon atoms. They are highly volatile and have low flavor thresholds, contributing significantly to the whole flavor in food processing [34]. Sixteen aldehydes were detected in the different cooked HB varieties, including hexanal, propanal, heptanal, acetaldehyde, (E)-2-heptenal, (E)-2-hexenal, and (E)-2-pentenal. These aldehydes likely play a major role in the flavor of the cooked HB. Alcohols primarily formed through lipid oxidation are known for their sweet, floral, and mild flavors [35]. A total of 10 alcohols were identified, primarily 1-octen-3-ol, 1-pentanol dimer, 1-penten-3-ol, 1-propanethiol, 3-methylbutan-1-ol dimer, and ethanol. In addition, among all alcohols, 1-octen-3-ol was thought to be produced by lipid oxidation during cooking and showed high content and low threshold with the smell of wild mushrooms, contributing the most to cooked HB aroma [36]. Ketones, known for their high flavor thresholds, are produced from the oxidation or thermal degradation of polyunsaturated fatty acids, the Maillard reaction, ester decomposition, and the breakdown of amino acids or microbial oxidation [37]. Nine ketones were found in the cooked HB samples, including 2-butanone, 1-penten-3-one, and 3-hydroxy-2-butanone dimer. These ketones contribute pleasant floral and fruity flavors with long-lasting fragrances, which may add to the overall flavor of cooked HB. Esters, typically formed by the esterification of low-molecular-weight fatty acids and alcohols, are known for their fruity flavors [38], making them important contributors to the fragrance of cooked HB. Seven esters were identified, including ethyl 2-methylbutanoate dimer, 2-methylbutanoic acid methyl ester, acetic acid ethyl ester, ethyl 2-methylpropionate, isovaleric acid methyl ester, butanoic acid ethyl ester, and ethyl lactate. These compounds likely enhance the fruity notes of cooked HB.

As displayed in Figure 3, the main volatile flavor compounds in different cooked HB varieties are largely similar, but there are notable differences in their relative contents. These discrepancies could be attributed to factors such as the origin of raw materials, barley variety, chemical composition, and other influences [39,40]. In the fingerprint analysis, compounds such as E-2-pentenal, nonanal, E-2-octenal, E-2-hexenal, E-2-heptenal, octanal, benzaldehyde, Z-4-heptenal, 1-pentene-3-ol, 1-octene-3-ol, butanol, amyl alcohol, 2-methyl-1-propanol, propanethiol, 2-butanone, and 2-heptanone were predominantly located in region A, representing the main flavor components of HB cooked from Chaiqing 1 (M1) and Beiqing 9 (M2) from Xining. Region B contained characteristic volatile compounds from HB made by Dalihuang (M7), Kunlun 14 (M9), Kunlun 15 (M10), Kunlun 17 (M11), and Kunlun 18 (M12) from Menyuan, as well as Chaiqing 1 (M13), Kunlun 14 (M14), and Kunlun 15 (M15) from Guinan. These included E-2-pentenal, nonanal, 4-methyl-3-penten-2-one, 3-methyl-2-pentenone, ethyl 2-methylbutyrate, ethyl 2-methylpropionate, and ethyl butyrate. The volatile compounds characterizing Beiqing 8 (M8) from Xining were primarily located in Region C, including butyraldehyde, Z-4-heptenal, benzaldehyde, 2-methylpropanal, 1-octene-3-ol, amyl alcohol, 2-methyl-1-propanol, propyl mercaptan, 2-butanone, 2-octanone, 2-heptanone, and ethyl lactate. Region D contained the characteristic volatile flavor compounds of HB cooked from Kunlun 16 (M16) and Kunlun 17 (M17) in Guinan, including E-2-pentenal, nonanal, E-2-octenal, E-2-hexenal, and E-2-heptenal. Overall, while the types of volatile compounds in different varieties of cooked HB were generally consistent, their relative contents varied, as a result, endowed with different flavor characteristics.

**Figure 4:**
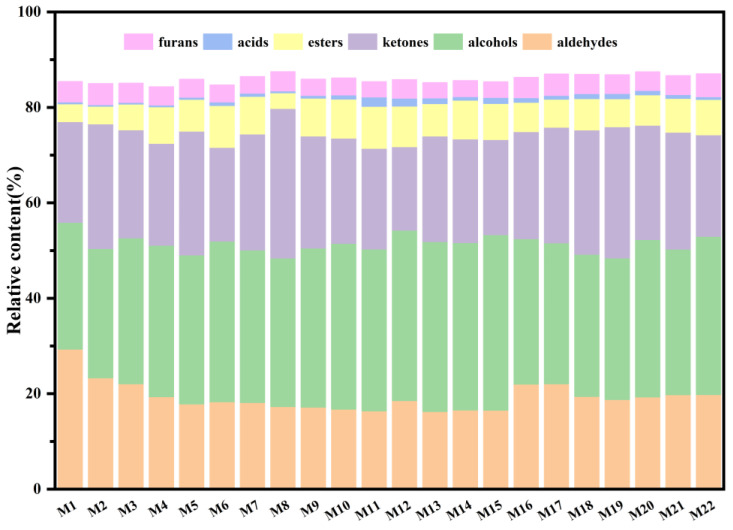
Contents of flavor compounds in cooked HB.

To further illustrate the distribution of flavor compounds in different cooked HB varieties, the relative contents of different flavor compounds were statistically analyzed and are shown in Figure 4. Alcohols made up a significant proportion in all cooked HB samples since alcohols are naturally occurring volatile flavor compounds in highland barley grains that are released during the cooking process. However, their contribution to the overall flavor may be limited due to their generally high flavor thresholds [41]. Additionally, the contents of furans and acids were relatively low across all samples. The aldehyde content was relatively high in the cooked HB prepared from Chaiqing 1 (M1) and Beiqing 9 (M2) from Xining, as well as Kunlun 16 (M16) and Kunlun 17 (M17) from Guinan. The content of ketones was generally higher in the cooked HB made from Beiqing 9 (M2) and Beiqing 8 (M8) from Xining, as well as Kunlun 14 (M18) and Kunlun 15 (M19) from Guide. Ester compounds were predominantly found in the cooked HB made from Kunlun 18 (M6) and Kunlun 15 (M10) from Xining, Kunlun 17 (M11) and Kunlun 18 (M12) from Menyuan, and Kunlun 14 (M14) from Guinan. These results aligned well with the findings from the fingerprint analysis and indicated that not only did the variety of raw materials affect its flavor compounds, but the planting region also had a certain impact, which was also consistent with the result of ANOVA.

### 3.5. Analysis of the Key Volatile Flavor Compounds of Cooked HB

To further study the effect of volatile compounds on the overall flavor, ROAV analysis was performed, and compounds with ROAV ≥ 0.1 were identified and summarized in Table 4. A total of 37 volatile compounds with ROAV ≥ 0.1 were detected, including 15 aldehydes, 6 alcohols, 8 ketones, 7 esters, and 1 furan. Among these, 13 aldehydes, 4 alcohols, 4 ketones, 7 esters, and 1 furan were common across all samples. The volatile compounds that contributed most to the overall flavor of cooked HB were hexanal and ethyl 2-methylbutanoate dimer, followed by 2-methylbutanoic acid methyl ester, 2-butanone, 1-octen-3-ol, 1-pentanol dimer, and 2-pentyl furan. Hexanal (ROAV = 100) was the dominant flavor component in HB cooked from Chaiqing 1 (M1), Beiqing 9 (M2), Doulihuang (M3), and 21Y-59 (M22) from Xining, Beiqing 8 (M8) from Menyuan, Kunlun 16 (M16) and Kunlun 17 (M17) from Guinan, Kunlun 14 (M18) and Kunlun 15 (M19) from Guide, and Chaiqing 1 (M20) and Kunlun 15 (M21) from Dulan. This result aligns with the findings of Wengang Zhang et al. [10]. Hexanal, known for its apple, vegetable, and fatty flavor, is produced through the lipoxygenase pathway as a degradation product of linoleic acid [42], which can offer a fresh, grassy-like flavor at relatively low concentration [43]. In contrast, for the HB prepared from Kunlun 14 (M4), Kunlun 15 (M5), and Kunlun 18 (M6) from Xining, Dulihuang (M7), Kunlun 14 (M9), Kunlun 15 (M10), Kunlun 17 (M11), and Kunlun 18 (M12) from Menyuan, as well as Kunlun 14 (M14) and Kunlun 15 (M15) from Guinan, the flavor substance with the highest ROAV value was ethyl 2-methylbutanoate dimer (ROAV = 100), which was considered to contribute the most to the overall flavor of the cooked HB mentioned above. However, there was limited information available specifically on the contribution of ethyl 2-methylbutanoate dimer to the flavor of HB.

Table 4 shows that the HB prepared from Kunlun 14 (M4) and Kunlun 18 (M6) in Xining had relatively high concentrations of ester flavor compounds, primarily ethyl 2-methylbutanoate dimer, 2-methylbutanoic acid methyl ester, and acetic acid ethyl ester. In contrast, aldehydes and alcohols such as hexanal, (E)-2-hexenal dimer, 1-octen-3-ol, and 1-pentanol dimer were more prevalent in HB made from Chaiqing (M1) in Xining. HB samples from Kunlun 14 (M18) and Kunlun 15 (M19) in Guide, Chai Qing 1 (M20) and Kunlun 15 (M21) in Dulan, and 21Y-59 Qing (M22) in Xining exhibited higher levels of ketones and esters, including 1-penten-3-one, 2-butanone, 3-hydroxy-2-butanone dimer, 2-methylbutanoic acid methyl ester, and acetic acid ethyl ester. Beiqing 9 (M2) from Xining and Beiqing 8 (M8) from Menyuan were richer in key volatile flavor compounds, especially aldehydes, alcohols, and ketones, such as hexanal, (E)-2-hexenal dimer, 1-octen-3-ol, 1-pentanol dimer, 1-penten-3-one, 2-butanone, and 3-hydroxy-2-butanone dimer. Additionally, Dulihuang (M3) in Xining was abundant in aldehydes, ketones, and esters like hexanal, (E)-2-hexenal dimer, 1-penten-3-one, 2-butanone, 3-hydroxy-2-butanone dimer, ethyl 2-methylbutanoate dimer, 2-methylbutanoic acid methyl ester, and acetic acid ethyl ester. These findings are consistent with the fingerprint and relative content analysis of key flavor compounds. A comprehensive analysis indicated that HB prepared from Beiqing 9 (M2) and Dulihuang (M3) in Xining, as well as Beiqing 8 (M8) in Menyuan, had a richer flavor profile and are more suitable for cooking. They were followed by HB from Kunlun 14 (M18) and Kunlun 15 (M19) in Guide, Chaiqing 1 (M20) and Kunlun 15 (M21) in Dulan, as well as Chaiqing 1 (M1), 21Y-59 (M22), Kunlun 14 (M4), and Kunlun 18 (M6) in Xining.

### 3.6. Cluster Analysis of Cooked HB

**Figure 5:**
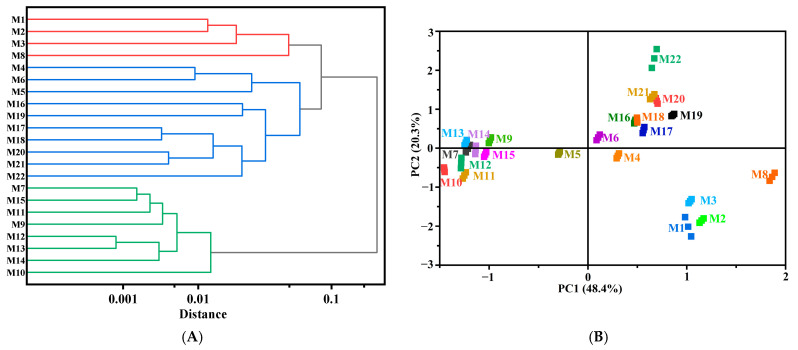
Hierarchical cluster analysis (**A**) and principal component analysis plot (**B**) of cooked HB. Note: samples with the same color had high similarity and were grouped together into the same category.

Hierarchical cluster analysis (HCA) and principal component analysis (PCA) plots of the 22 cooked HB varieties, conducted by integrating cooking quality, sensory scores, and key flavor substances, are presented in Figure 5. The 22 cooked HB were clustered based on PC1 and PC2, which account for 48.4% and 20.3% of the explained variance of the data, respectively. The 22 cooked HB could be mainly divided into three categories combining cluster analysis with principal component analysis. Category 1 consisted of four samples: Chaiqing 1 (M1), Beiqing 9 (M2), and Doulihuang (M3) from Xining, and Beiqing 8 (M8) from Menyuan. These samples shared similar key flavor substances but had relatively lower sensory scores. Category 2 included Kunlun 14 (M4), Kunlun 15 (M5), and Kunlun 18 (M6) from Xining with relatively poor cooking quality, as well as Kunlun 16 (M16) and Kunlun 17 (M17) from Guinan, Kunlun 14 (M18) and Kunlun 15 (M19) from Guide, and Chaiqing 1 (M20) and Kunlun 15 (M21) from Dulan. These samples had higher cooking quality, sensory scores, and relative abundance of key flavor substances, making them the most suitable varieties for cooking. The only waxy highland barley 21Y-59 (M22) from Xining was also assigned to Group 2, which possessed the highest water absorption rate but relatively moderate quality in other aspects. Category 3 comprised Dulihuang (M7), Kunlun 14 (M9), and Kunlun 17 (M11) from Menyuan, and Kunlun 15 (M15) from Guinan. These samples showed good cooking quality but had a limited variety of key flavor substances. Kunlun 15 (M10) and Kunlun 18 (M12) from Menyuan, and Chaiqing 1 (M13) and Kunlun 14 (M14) from Guinan were also placed in Group 3 for their relatively high sensory scores and cooking quality, making them more suitable for cooking. HCA and PCA provided a comprehensive evaluation of the quality of different HB varieties, allowing for the identification of HB varieties those are most suitable for cooking.

## 4. Conclusions

In this study, the effects of 22 different types of highland barley raw materials (including 10 common varieties and 5 main cultivation areas in the Qinghai Province) on the comprehensive edible quality and volatile flavor components of HB were investigated. All cooked HB materials exhibited a unique flavor, firm texture, full and transparent appearance, and a texture that did not stick. However, there were notable differences in both the overall edible quality and flavor characteristics. The highest volume expansion rate was observed in HB from Guinan (average 126.73%), while the highest water absorption rate was found in HB from Menyuan (average 62.52%). In terms of sensory evaluation, Kunlun 15 (M5) from Xining, Kunlun 14 (M9) from Menyuan, Chaiqing 1 (M13) and Kunlun (M14) from Guinan, and Chaiqing 1 (M20) and Kunlun 15 (M21) from Dulan performed relatively better than other varieties. The main flavor components of boiled HB were primarily aldehydes, esters, and ketones, including hexanal, (E)-2-hexenal dimer, ethyl 2-methylbutanoate dimer, 2-methylbutanoic acid methyl ester, acetic acid ethyl ester, ethyl 2-methylpropionate, 2-butanone, and 1-penten-3-one. Based on a comprehensive analysis, it was found that Kunlun 16 (M16) and Kunlun 17 (M17) from Guinan, Kunlun 14 (M18) and Kunlun 15 (M19) from Guide, and Chaiqing 1 (M20) and Kunlun 15 (M21) from Dulan had the highest overall quality, which makes them more suitable for cooking. Followed by Kunlun 15 (M10) and Kunlun 18 (M12) from Menyuan, and Chaiqing 1 (M13) and Kunlun 14 (M14) from Guinan. This study provides valuable theoretical support for the development and scientific processing of high-quality HB products. In this study, the influence of the main varieties as well as the main cultivation areas of HB on the processing quality of cooked HB were mainly investigated. It was worth noting that environment and variety factors, as well as their interactions, all had a significant impact on the nutritional quality of highland barley, especially environment factors, which means that it is very important to screen suitable varieties for processing from the main cultivated varieties planted in different regions. However, whether cultivation measures and processing methods have an impact on the processing quality of HB still need to be further investigated.

## Figures and Tables

**Table 1:** Sample information.

No.	Varieties	Regions	Altitude (m)	Average Annual Temperature (°C)	Annual Precipitation (mm)	Annual Sunshine Duration (h)	No.	Varieties	Regions	Altitude (m)	Average Annual Temperature (°C)	Annual Precipitation (mm)	Annual Sunshine Duration (h)
M1	Chaiqing 1	Xining	2300	6.1	431.2	2392	M12	Kunlun 18	Menyuan	2850	2.2	504.5	2357
M2	Beiqing 9	Xining	2300	6.1	431.2	2392	M13	Ziqing 1	Guinan	3120	2.5	466.0	2254
M3	Dulihuang	Xining	2300	6.1	431.2	2392	M14	Kunlun 14	Guinan	3120	2.5	466.0	2254
M4	Kunlun 14	Xining	2300	6.1	431.2	2392	M15	Kunlun 15	Guinan	3120	2.5	466.0	2254
M5	Kunlun 15	Xining	2300	6.1	431.2	2392	M16	Kunlun 16	Guinan	3120	2.5	466.0	2254
M6	Kunlun 18	Xining	2300	6.1	431.2	2392	M17	Kunlun 17	Guinan	3120	2.5	466.0	2254
M7	Dulihuang	Menyuan	2850	2.2	504.5	2357	M18	Kunlun 14	Guide	2238	8.8	266.9	2505
M8	Beiqing 8	Menyuan	2850	2.2	504.5	2357	M19	Kunlun 15	Guide	2238	8.8	266.9	2505
M9	Kunlun 14	Menyuan	2850	2.2	504.5	2357	M20	Chaiqing 1	Dulan	3190	3.7	175.9	2805
M10	Kunlun 15	Menyuan	2850	2.2	504.5	2357	M21	Kunlun 15	Dulan	3190	3.7	175.9	2805
M11	Kunlun 17	Menyuan	2850	2.2	504.5	2357	M22	21Y-59	Xining	2300	6.1	431.2	2392

Abbreviations: No., number; M1–M22 are the numbers of 22 different highland barley raw materials.

**Table 2:** Sensory scoring table.

Evaluation Indicators	Mark	Assessment Criteria
Smell (20)	17–20	Processes the unique fragrance of cooked HB and rich aroma
	13–16	Processes the unique fragrance of cooked HB and light aroma
	7–12	The pleasant smell is not obvious, but no off odor
	0–6	Have a slight odor
Color (15)	11–15	Yellow or white, uniform, shiny
	6–10	The color is normal, slightly uneven, slightly shiny
	0–5	The color is dark or heterochromatic, dull
Appearance structure (10)	8–10	The grain structure is tight and the integrity is good
	5–7	The structure of most HB grains is tight and complete, and a few grains are cracked
	0–4	The size uniformity is poor, and a small number of rice grains appear exploded
Palatability (viscosity, elasticity, hardness) (30)	26–30	Smooth and chewy, good viscoelasticity, not sticking to teeth, moderate hardness
	21–25	Slightly chewy, viscoelastic slightly poor, not sticking toteeth, slightly hard or soft
	16–20	Rice is loose and not smooth, poor viscoelasticity, relatively hard or soft
	0–15	Rice grains are stiff, poor viscoelasticity, large viscosity, too hard or too soft
Taste (25)	23–25	It tastes strongly aromatic and sweet when chewing
	18–22	It tastes a light strongly aromatic and sweet when chewing
	15–17	It tastes not aromatic and sweet but a little bitter when chewing
	0–14	It tastes not aromatic and sweet but a little bitter and odor when chewing

**Table 3:** Analysis of texture characteristics of the cooked HB.

No.	Hardness/N	Adhesiveness/mJ	Cohesiveness	Elasticity/mm	Stickiness/N	Chewiness/mJ
M1	17.93 ± 3.34 ^cde^	0.006 ± 0.002 ^bc^	0.65 ± 0.03 ^bc^	0.73 ± 0.03 ^fgh^	11.65 ± 2.71 ^cd^	8.505 ± 2.304 ^efgh^
M2	17.99 ± 1.42 ^cde^	0.005 ± 0.001 ^bc^	0.66 ± 0.02 ^bc^	0.78 ± 0.06 ^bcdefg^	11.88 ± 0.92 ^cd^	9.297 ± 1.158 ^cdefg^
M3	14.94 ± 4.18 ^defg^	0.007 ± 0.001 ^bc^	0.67 ± 0.03 ^bc^	0.66 ± 0.06 ^hi^	9.98 ± 2.79 ^def^	6.665 ± 2.39 ^fghij^
M4	23.86 ± 1.58 ^b^	0.008 ± 0.002 ^bc^	0.66 ± 0.04 ^bc^	0.81 ± 0.06 ^bcde^	15.79 ± 1.68 ^b^	12.67 ± 0.803 ^b^
M5	13.49 ± 3.15 ^efgh^	0.008 ± 0.001 ^bc^	0.63 ± 0.07 ^b^	0.71 ± 0.03 ^gh^	8.40 ± 1.08 ^efgh^	5.895 ± 0.745 ^hijk^
M6	13.49 ± 1.76 ^efgh^	0.007 ± 0.001 ^bc^	0.66 ± 0.02 ^bc^	0.71 ± 0.06 ^gh^	8.89 ± 1.46 ^defg^	6.327 ± 1.509 ^ghijk^
M7	8.62 ± 0.72 ^hij^	0.006 ± 0.002 ^bc^	0.69 ± 0.05 ^bc^	0.68 ± 0.06 ^hi^	5.92 ± 0.59 ^gh^	4.04 ± 0.694 ^jk^
M8	11.67 ± 1.52 ^ghij^	0.008 ± 0.001 ^bc^	0.66 ± 0.04 ^bc^	0.76 ± 0.03 ^efg^	7.76 ± 1.33 ^fgh^	5.897 ± 1.165 ^hijk^
M9	9.08 ± 0.87 ^hij^	0.008 ± 0.001 ^bc^	0.66 ± 0.04 ^bc^	0.72 ± 0.02 ^gh^	5.96 ± 0.89 ^gh^	4.282 ± 0.738 ^ijk^
M10	12.42 ± 1.52 ^fghij^	0.009 ± 0.003 ^b^	0.68 ± 0.02 ^bc^	0.77 ± 0.04 ^cdefg^	8.40 ± 0.83 ^efgh^	6.495 ± 0.836 ^fghij^
M11	7.89 ± 0.71 ^j^	0.0056 ± 0.002 ^c^	0.67 ± 0.03 ^bc^	0.61 ± 0.04 ^i^	5.33 ± 0.60 ^h^	3.285 ± 0.568 ^k^
M12	11.13 ± 2.38 ^ghij^	0.006 ± 0.001 ^bc^	0.70 ± 0.03 ^c^	0.78 ± 0.03 ^cdefg^	7.79 ± 1.81 ^fgh^	6.057 ± 1.562 ^hijk^
M13	11.79 ± 1.55 ^ghij^	0.007 ± 0.001 ^bc^	0.67 ± 0.03 ^bc^	0.84 ± 0.05 ^bcd^	7.84 ± 0.81 ^fgh^	6.54 ± 0.904 ^fghij^
M14	8.40 ± 3.79 ^ij^	0.008 ± 0.002 ^bc^	0.66 ± 0.05 ^bc^	0.82 ± 0.03 ^bcde^	7.18 ± 1.07 ^fgh^	5.87 ± 0.829 ^hijk^
M15	10.63 ± 0.83 ^ghij^	0.005 ± 0.001 ^bc^	0.66 ± 0.01 ^bc^	0.71 ± 0.02 ^gh^	7.02 ± 0.45 ^fgh^	4.962 ± 0.422 ^ijk^
M16	17.27 ± 2.42 ^cdef^	0.005 ± 0.001 ^c^	0.67 ± 0.01 ^bc^	0.76 ± 0.01 ^defg^	11.47 ± 1.71 ^cd^	8.757 ± 1.324 ^defgh^
M17	13.31 ± 2.39 ^efghi^	0.006 ± 0.001 ^bc^	0.68 ± 0.04 ^bc^	0.80 ± 0.05 ^bcdef^	9.10 ± 2.25 ^def^	7.31 ± 1.972 ^fghi^
M18	19.63 ± 2.53 ^bcd^	0.006 ± 0.001 ^bc^	0.67 ± 0.02 ^bc^	0.81 ± 0.01 ^bcde^	13.23 ± 1.41 ^bc^	10.777 ± 1.289 ^bcde^
M19	16.69 ± 2.34 ^cdef^	0.006 ± 0.001 ^bc^	0.68 ± 0.03 ^bc^	0.85 ± 0.06 ^b^	11.33 ± 1.95 ^cde^	9.575 ± 0.958 ^cdef^
M20	20.03 ± 6.01 ^bc^	0.005 ± 0.002 ^c^	0.69 ± 0.02 ^bc^	0.85 ± 0.05 ^bc^	13.73 ± 4.08 ^bc^	11.685 ± 3.645 ^bcd^
M21	21.13 ± 3.73 ^bc^	0.005 ± 0.001 ^c^	0.68 ± 0.04 ^bc^	0.85 ± 0.03 ^b^	14.19 ± 2.09 ^bc^	12.06 ± 1.434 ^bc^
M22	40.12 ± 6.95 ^a^	0.014 ± 0.005 ^a^	0.51 ± 0.04 ^a^	1.17 ± 0.10 ^a^	20.33 ± 3.34 ^a^	24.11 ± 5.809 ^a^

Abbreviations: No., number. Note: all data were expressed by mean ± standard deviation. Different superscript lowercase letters in the same row indicate statistical differences between samples at *p* < 0.05 level.

**Table 4:** Analysis of key volatile flavor compounds in different cooked HB.

**Compounds**		**Aldehydes (15)**														
		**(E)-2-Octenal Dimer**	**(Z)-4-Heptenal**	**(E)-2-Heptenal Dimer**	**(E)-2-Hexenal Dimer**	**(E)-2-Pentenal Dimer**	**1-Octanal Dimer**	**2-Methyl Propanal**	**3-Methyl Butanal**	**Acetaldehyde**	**Butanal**	**Heptaldehyde Dimer**	**Hexanal**	**Nonanal Dimer**	**n-Pentanal**	**Propanal Dimer**
RI		1420.0	1250.0	1333.2	1229.3	1146.3	1299.0	842.3	934.2	819.7	925.3	1195.1	1099.9	1401.0	1003.3	840.5
RT		1057.6	727.5	855.2	698.5	561.3	799.2	266.0	321.8	253.9	315.9	653.8	483.8	984.0	372.9	265.1
DT		1.8	1.2	1.7	1.5	1.4	1.8	1.3	1.4	1.0	1.3	1.7	1.6	1.9	1.4	1.2
OT (µg/kg)		49	2.8	12	5	55	3.4	2.1	240	15	9	2.8	2.4	2.8	20	9.5
Odorant Description		Flower, fat, meat	Burnt, sweet, milk	Fat	Fruity, grassy	Fruity	Mold	Fruity	Spicy, thin and fruity	——	Dilute with a clear fragrance	Fruity	Apple, grassy, fat	Rose, citrus	Almond fragrance	——
ROAV	M1	1.39 ± 0.04 ^a^	2.80 ± 0.24 ^e^	16.29 ± 0.08 ^a^	14.60 ± 1.15 ^a^	1.08 ± 0.24 ^c^	2.03 ± 0.00 ^a^	0.60 ± 0.00 ^i^	0.12 ± 0.00 ^d^	1.44 ± 0.02 ^k^	1.91 ± 0.00 ^f^	10.07 ± 1.42 ^i^	100.00 ± 0.00 ^a^	7.05 ± 0.01 ^a^	2.22 ± 0.02 ^b^	8.64 ± 0.32 ^g^
	M2	1.26 ± 0.05 ^b^	3.83 ± 0.11 ^bc^	11.00 ± 0.07 ^b^	13.53 ± 1.30 ^c^	1.20 ± 0.08 ^b^	1.85 ± 0.06 ^b^	0.88 ± 0.01 ^def^	0.11 ± 0.00 ^ef^	2.02 ± 0.01 ^h^	2.22 ± 0.00 ^e^	9.77 ± 0.56 ^i^	100.00 ± 0.00 ^a^	7.00 ± 0.01 ^a^	1.57 ± 0.01 ^d^	10.00 ± 0.75 ^de^
	M3	0.68 ± 0.01 ^d^	3.81 ± 0.25 ^bc^	9.69 ± 0.07 ^c^	12.49 ± 0.70 ^d^	1.08 ± 0.01 ^c^	1.40 ± 0.01 ^c^	1.13 ± 0.06 ^b^	0.16 ± 0.01 ^b^	2.85 ± 0.56 ^cd^	3.56 ± 0.00 ^b^	18.92 ± 1.29 ^a^	100.00 ± 0.00 ^a^	5.37 ± 0.01 ^c^	1.76 ± 0.00 ^c^	11.03 ± 1.27 ^b^
	M4	0.36 ± 0.01 ^jk^	2.90 ± 0.15 ^e^	2.57 ± 0.04 ^gh^	7.41 ± 0.82 ^i^	0.81 ± 0.01 ^f^	0.67 ± 0.01 ^fg^	0.77 ± 0.01 ^g^	0.11 ± 0.00 ^f^	2.56 ± 0.10 ^e^	4.51 ± 0.03 ^a^	12.07 ± 0.98 ^g^	95.2 ± 2.25 ^b^	2.55 ± 0.01 ^gh^	0.60 ± 0.02 ^i^	9.44 ± 0.78 ^ef^
	M5	0.37 ± 0.04 ^j^	2.23 ± 0.99 ^f^	2.03 ± 0.85 ^hi^	6.66 ± 0.76 ^j^	0.62 ± 0.11 ^h^	0.61 ± 0.11 ^g^	0.50 ± 0.12 ^j^	0.07 ± 0.01 ^g^	1.94 ± 0.07 ^i^	2.83 ± 0.42 ^d^	9.94 ± 0.71 ^i^	63.81 ± 1.50 ^d^	2.04 ± 0.71 ^i^	0.57 ± 0.06 ^ij^	6.92 ± 1.15 ^h^
	M6	0.34 ± 0.02 ^k^	2.90 ± 0.24 ^e^	2.00 ± 0.06 ^i^	6.11 ± 0.90 ^k^	0.72 ± 0.04 ^g^	0.86 ± 0.01 ^d^	1.08 ± 0.28 ^b^	0.11 ± 0.00 ^ef^	2.80 ± 0.16 ^d^	4.48 ± 0.04 ^a^	11.84 ± 0.01 ^gh^	87.89 ± 1.39 ^c^	2.32 ± 0.01 ^hi^	0.54 ± 0.01 ^j^	9.51 ± 0.56 ^ef^
	M7	0.14 ± 0.01 ^lm^	1.07 ± 0.09 ^hi^	0.81 ± 0.01 ^j^	3.90 ± 0.04 ^lm^	0.30 ± 0.01 ^i^	0.27 ± 0.00 ^j^	0.37 ± 0.01 ^k^	0.03 ± 0.00 ^ij^	1.01 ± 0.01 ^n^	1.59 ± 0.03 ^g^	4.64 ± 0.42 ^l^	34.45 ± 0.64 ^f^	0.88 ± 0.01 ^j^	0.29 ± 0.01 ^l^	3.54 ± 0.62 ^j^
	M8	0.46 ± 0.02 ^h^	8.82 ± 1.14 ^a^	6.21 ± 0.04 ^d^	14.60 ± 0.54 ^a^	0.92 ± 0.01 ^e^	0.62 ± 0.01 ^g^	4.50 ± 0.15 ^a^	0.23 ± 0.01 ^a^	3.85 ± 0.13 ^a^	2.68 ± 0.03 ^d^	13.32 ± 0.01 ^e^	100.00 ± 0.00 ^a^	3.56 ± 0.42 ^de^	2.99 ± 0.08 ^a^	12.38 ± 1.42 ^a^
	M9	0.14 ± 0.01 ^lm^	1.39 ± 0.03 ^gh^	0.76 ± 0.01 ^jk^	5.98 ± 0.08 ^k^	0.29 ± 0.01 ^i^	0.24 ± 0.01 ^j^	0.62 ± 0.06 ^hi^	0.05 ± 0.01 ^h^	1.24 ± 0.01 ^l^	2.04 ± 0.06 ^ef^	4.99 ± 0.84 ^k^	38.59 ± 0.42 ^e^	0.87 ± 0.04 ^j^	0.33 ± 0.02 ^k^	4.38 ± 0.37 ^i^
	M10	0.05 ± 0.01 ^o^	0.56 ± 0.02 ^j^	0.25 ± 0.03 ^l^	2.34 ± 0.02 ^o^	0.11 ± 0.01 ^l^	0.11 ± 0.01 ^k^	0.19 ± 0.01 ^m^	0.02 ± 0.01 ^l^	0.54 ± 0.08 ^q^	0.84 ± 0.05 ^i^	2.14 ± 0.01 ^o^	14.68 ± 0.95 ^k^	0.35 ± 0.01 ^k^	0.13 ± 0.02 ^o^	1.77 ± 0.04 ^l^
	M11	0.09 ± 0.01 ^n^	1.08 ± 0.01 ^hi^	0.36 ± 0.01 ^kl^	2.19 ± 0.04 ^o^	0.14 ± 0 ^kl^	0.22 ± 0.01 ^j^	0.77 ± 0.01 ^g^	0.05 ± 0.01 ^h^	0.77 ± 0.10 ^p^	1.46 ± 0.00 ^g^	4.33 ± 0.05 ^lm^	25.14 ± 1.85 ^i^	0.66 ± 0.04 ^jk^	0.21 ± 0.04 ^n^	2.58 ± 0.00 ^k^
	M12	0.09 ± 0.00 ^n^	0.86 ± 0.02 ^ij^	0.54 ± 0.01 ^jkl^	3.31 ± 0.01 ^n^	0.20 ± 0.00 ^jk^	0.25 ± 0.00 ^j^	0.35 ± 0.01 ^k^	0.02 ± 0.01 ^kl^	0.91 ± 0.02 ^o^	1.07 ± 0.01 ^h^	3.42 ± 0.01 ^n^	23.79 ± 0.98 ^j^	0.66 ± 0.01 ^jk^	0.19 ± 0.00 ^n^	2.65 ± 0.00 ^k^
	M13	0.10 ± 0.02 ^n^	0.97 ± 0.02 ^hij^	0.51 ± 0.01 ^jkl^	3.76 ± 0.34 ^m^	0.22 ± 0.00 ^j^	0.28 ± 0.01 ^j^	0.28 ± 0.01 ^l^	0.02 ± 0.01 ^kl^	1.19 ± 0.01 ^m^	1.08 ± 0.00 ^h^	4.09 ± 0.01 ^m^	25.36 ± 0.71 ^i^	0.68 ± 0.08 ^jk^	0.19 ± 0.00 ^n^	3.2 ± 0.00 ^j^
	M14	0.11 ± 0.02 ^mn^	1.16 ± 0.01 ^hi^	0.60 ± 0.00 ^jkl^	4.19 ± 0.06 ^l^	0.22 ± 0.01 ^j^	0.27 ± 0.01 ^j^	0.37 ± 0.01 ^k^	0.03 ± 0.00 ^jk^	1.18 ± 0.10 ^m^	1.40 ± 0.01 ^g^	4.27 ± 0.03 ^m^	29.25 ± 1.06 ^h^	0.74 ± 0.01 ^j^	0.24 ± 0.01 ^m^	3.38 ± 0.00 ^j^
	M15	0.16 ± 0.02 ^l^	1.68 ± 0.06 ^g^	0.73 ± 0.03 ^jk^	4.13 ± 0.08 ^lm^	0.28 ± 0.01 ^i^	0.43 ± 0.01 ^i^	0.62 ± 0.01 ^hi^	0.05 ± 0.01 ^hi^	1.70 ± 0.01 ^j^	1.48 ± 0.01 ^g^	5.92 ± 0.28 ^j^	33.45 ± 0.85 ^g^	0.99 ± 0.04 ^j^	0.35 ± 0.00 ^k^	4.58 ± 0.00 ^i^
	M16	0.62 ± 0.00 ^e^	4.10 ± 0.07 ^b^	4.02 ± 0.59 ^f^	10.57 ± 0.60 ^f^	1.09 ± 0.01 ^c^	0.77 ± 0.08 ^e^	0.94 ± 0.01 ^cd^	0.14 ± 0.02 ^c^	2.56 ± 0.01 ^e^	2.85 ± 0.02 ^d^	13.6 ± 0.42 ^e^	100.00 ± 0.00 ^a^	3.87 ± 0.03 ^d^	1.07 ± 0.04 ^h^	9.47 ± 0.00 ^ef^
	M17	0.93 ± 0.00 ^c^	3.40 ± 0.14 ^cd^	5.49 ± 0.48 ^e^	14.94 ± 0.66 ^a^	1.26 ± 0.01 ^a^	0.52 ± 0.01 ^h^	0.84 ± 0.01 ^ef^	0.11 ± 0.00 ^ef^	2.30 ± 0.03 ^g^	3.09 ± 0.01 ^c^	11.71 ± 0.70 ^h^	100.00 ± 0.00 ^a^	5.79 ± 0.09 ^b^	1.43 ± 0.01 ^e^	9.53 ± 0.00 ^ef^
	M18	0.42 ± 0.02 ^i^	3.77 ± 0.32 ^bc^	2.18 ± 0.06 ^hi^	8.65 ± 0.75 ^h^	0.75 ± 0.02 ^fg^	0.70 ± 0.01 ^f^	0.97 ± 0.01 ^c^	0.13 ± 0.02 ^cd^	2.88 ± 0.01 ^c^	3.14 ± 0.00 ^c^	13.98 ± 0.05 ^d^	100.00 ± 0.00 ^a^	3.04 ± 0.01 ^f^	1.09 ± 0.07 ^h^	9.46 ± 0.01 ^ef^
	M19	0.50 ± 0.03 ^g^	3.29 ± 0.69 ^de^	2.95 ± 0.02 ^g^	9.63 ± 0.73 ^g^	0.92 ± 0.01 ^e^	0.62 ± 0.01 ^g^	0.67 ± 0.00 ^h^	0.08 ± 0.02 ^g^	2.38 ± 0.01 ^f^	3.23 ± 0.01 ^c^	13.65 ± 0.91 ^e^	100.00 ± 0.00 ^a^	3.54 ± 0.03 ^de^	1.06 ± 0.01 ^h^	9.2 ± 0.00 ^f^
	M20	0.45 ± 0.04 ^h^	3.17 ± 0.82 ^de^	2.87 ± 0.01 ^g^	14.00 ± 0.75 ^b^	1.01 ± 0.01 ^d^	0.65 ± 0.01 ^fg^	0.88 ± 0.01 ^def^	0.13 ± 0.02 ^cd^	2.81 ± 0.01 ^d^	3.17 ± 0.01 ^c^	12.83 ± 0.08 ^f^	100.00 ± 0.00 ^a^	2.78 ± 0.04 ^fg^	1.13 ± 0.02 ^g^	10.41 ± 0.01 ^cd^
	M21	0.53 ± 0.03 ^f^	4.15 ± 0.95 ^b^	3.99 ± 0.30 ^f^	9.88 ± 1.09 ^g^	1.04 ± 0.01 ^cd^	0.77 ± 0.01 ^e^	0.89 ± 0.01 ^de^	0.17 ± 0.02 ^b^	2.87 ± 0.01 ^c^	3.62 ± 0.00 ^b^	15.51 ± 0.01 ^b^	100.00 ± 0.00 ^a^	3.48 ± 0.03 ^e^	1.14 ± 0.03 ^fg^	10.56 ± 0.01 ^bc^
	M22	0.42 ± 0.02 ^i^	1.74 ± 0.08 ^g^	2.44 ± 0.05 ^h^	11.91 ± 0.62 ^e^	0.75 ± 0.01 ^g^	0.82 ± 0.01 ^de^	0.83 ± 0.01 ^fg^	0.12 ± 0.01 ^de^	3.55 ± 0.01 ^b^	3.25 ± 0.01 ^c^	15.05 ± 0.05 ^c^	100.00 ± 0.00 ^a^	3.73 ± 0.02 ^de^	1.17 ± 0.03 ^f^	9.83 ± 0.00 ^e^
**Compounds**		**Alcohols (6)**							**Ketones (8)**							
		**1-Octen-3-ol**	**1-Pentanol Dimer**	**1-Penten-3-ol**	**1-Propanethiol**	**3-Methylbutan-1-ol Dimer**		**Ethanol**	**1-Penten-3-One**	**2-Butanone**	**2-Heptanone Dimer**	**2-Octanone**	**2-Pentanone**	**3-Hydroxy-2-Butanone Dimer**	**3-Methyl-2-Pentanone**	**3-Penten-2-One, 4-Methyl**
RI		1444.5	1262.0	1171.9	854.1	1216.8		950.1	1029.2	924.1	1190.9	1291.8	1001.0	1297.7	1015.3	1131.7
RT		1160.8	744.0	609.5	272.6	681.7		332.5	399.7	315.1	647.7	787.9	370.7	797.0	385.1	535.7
DT		1.2	1.5	0.9	1.4	1.5		1.1	1.1	1.3	1.6	1.8	1.4	1.3	1.5	1.5
OT (µg/kg)		1.5	5	358.1	3.1	291		8000	1.3	3	410	41	1380	14	41	9
Odorant Description		Soil, oil, floral, mold, mushroom	Bread, wine, fruit	Fruity	——	——		——	Citrus, vanilla	Vanilla	Fruity	Mold, milk, cheese, mushroom	——	Sweet, dairy flavor	Fruity	Floral and fruity
ROAV	M1	23.60 ± 0.69 ^b^	27.45 ± 1.56 ^b^	0.16 ± 0.01 ^d^	1.11 ± 0.05 ^d^	0.12 ± 0.01 ^m^		0.06 ± 0.00 ^e^	8.19 ± 1.00 ^l^	36.66 ± 0.48 ^d^	0.26 ± 0.07 ^c^	0.01 ± 0.02 ^de^	0.05 ± 0.02 ^d^	2.13 ± 0.07 ^j^	0.11 ± 0.01 ^ij^	0.52 ± 0.01 ^h^
	M2	22.19 ± 1.37 ^c^	27.88 ± 1.08 ^b^	0.19 ± 0.01 ^c^	1.91 ± 0.04 ^b^	0.13 ± 0.01 ^lm^		0.07 ± 0.01 ^cd^	10.24 ± 1.00 ^i^	53.62 ± 1.84 ^b^	0.45 ± 0.01 ^b^	0.02 ± 0.00 ^c^	0.10 ± 0.02 ^a^	3.06 ± 0.15 ^ij^	0.53 ± 0.01 ^a^	0.58 ± 0.00 ^fg^
	M3	22.14 ± 1.20 ^c^	16.53 ± 0.59 ^c^	0.2 ± 0.01 ^b^	1.53 ± 0.04 ^c^	0.12 ± 0.01 ^lm^		0.1 ± 0.00 ^a^	13.08 ± 0.70 ^b^	42.44 ± 0.42 ^c^	0.25 ± 0.01 ^c^	0.02 ± 0.01 ^c^	0.08 ± 0.00 ^b^	4.18 ± 0.56 ^i^	0.32 ± 0.01 ^c^	0.44 ± 0.01 ^i^
	M4	9.97 ± 1.10 ^e^	3.71 ± 0.07 ^gh^	0.11 ± 0.01 ^e^	0.94 ± 0.04 ^e^	0.13 ± 0.01 ^lm^		0.09 ± 0.00 ^b^	10.75 ± 0.14 ^g^	26.22 ± 0.99 ^f^	0.16 ± 0.02 ^efgh^	0.02 ± 0.02 ^c^	0.06 ± 0.01 ^c^	3.25 ± 0.49 ^ij^	0.16 ± 0.01 ^f^	0.54 ± 0.06 ^gh^
	M5	5.20 ± 0.27 ^j^	3.12 ± 0.77 ^hi^	0.06 ± 0.01 ^j^	0.59 ± 0.08 ^i^	0.29 ± 0.06 ^g^		0.06 ± 0.01 ^e^	7.60 ± 0.11 ^m^	18.89 ± 0.85 ^i^	0.14 ± 0.06 ^gh^	0.01 ± 0.01 ^cd^	0.04 ± 0.01 ^de^	12.70 ± 0.11 ^e^	0.16 ± 0.01 ^f^	0.64 ± 0.07 ^e^
	M6	9.53 ± 0.57 ^ef^	3.25 ± 0.18 ^ghi^	0.09 ± 0.01 ^gh^	0.73 ± 0.07 ^g^	0.17 ± 0.01 ^jk^		0.09 ± 0.00 ^b^	11.25 ± 0.99 ^e^	21.67 ± 2.12 ^gh^	0.12 ± 0.01 ^h^	0.02 ± 0.03 ^c^	0.04 ± 0.00 ^ef^	5.78 ± 0.29 ^h^	0.1 ± 0.02 ^ij^	0.61 ± 0.01 ^ef^
	M7	3.47 ± 0.22 ^k^	1.79 ± 0.10 ^kl^	0.03 ± 0.00 ^k^	0.25 ± 0.01 ^k^	0.21 ± 0.07 ^i^		0.03 ± 0.00 ^g^	4.00 ± 0.51 ^p^	7.97 ± 0.57 ^l^	0.06 ± 0.07 ^ij^	0.00 ± 0.00 ^f^	0.02 ± 0.01 ^g^	7.49 ± 0.29 ^g^	0.12 ± 0.01 ^gh^	0.35 ± 0.02 ^j^
	M8	33.61 ± 0.42 ^a^	36.24 ± 1.07 ^a^	0.22 ± 0.01 ^a^	4.07 ± 0.34 ^a^	0.82 ± 0.07 ^a^		0.09 ± 0.00 ^b^	15.35 ± 0.92 ^a^	61.27 ± 4.38 ^a^	0.77 ± 0.01 ^a^	0.12 ± 0.00 ^a^	0.10 ± 0.00 ^a^	36.87 ± 0.79 ^a^	0.54 ± 0.01 ^a^	1.69 ± 0.01 ^a^
	M9	3.58 ± 0.53 ^k^	2.70 ± 0.33 ^ij^	0.03 ± 0.01 ^kl^	0.31 ± 0.04 ^j^	0.35 ± 0.04 ^f^		0.04 ± 0.00 ^f^	4.38 ± 0.32 ^o^	7.75 ± 0.85 ^l^	0.07 ± 0.06 ^i^	0.01 ± 0.00 ^de^	0.02 ± 0.01 ^g^	10.18 ± 0.59 ^f^	0.28 ± 0.01 ^d^	0.52 ± 0.01 ^h^
	M10	1.82 ± 0.01 ^m^	1.39 ± 0.06 ^lm^	0.01 ± 0.00 ^m^	0.08 ± 0.00 ^n^	0.15 ± 0.01 ^kl^		0.01 ± 0.00 ^i^	1.60 ± 0.19 ^t^	3.38 ± 0.16 ^n^	0.02 ± 0.03 ^k^	0.00 ± 0.01 ^f^	0.01 ± 0.02 ^h^	3.01 ± 0.66 ^ij^	0.13 ± 0.01 ^g^	0.25 ± 0.01 ^k^
	M11	3.68 ± 0.25 ^k^	1.04 ± 0.37 ^m^	0.02 ± 0.00 ^lm^	0.21 ± 0.01 ^l^	0.17 ± 0.01 ^jk^		0.03 ± 0.00 ^g^	3.58 ± 0.58 ^q^	5.74 ± 0.42 ^m^	0.03 ± 0.04 ^jk^	0.01 ± 0.01 ^ef^	0.01 ± 0.01 ^h^	6.37 ± 0.17 ^gh^	0.04 ± 0.02 ^m^	0.23 ± 0.01 ^k^
	M12	3.53 ± 0.46 ^k^	1.82 ± 0.76 ^kl^	0.02 ± 0.00 ^lm^	0.15 ± 0.01 ^m^	0.20 ± 0.01 ^ij^		0.02 ± 0.00 ^h^	2.64 ± 0.08 ^s^	5.39 ± 0.39 ^m^	0.04 ± 0.02 ^ijk^	0.00 ± 0.02 ^f^	0.01 ± 0.00 ^h^	2.12 ± 0.11 ^j^	0.09 ± 0.02 ^jk^	0.36 ± 0.01 ^j^
	M13	2.84 ± 0.07 ^l^	2.03 ± 0.67 ^jkl^	0.02 ± 0.00 ^lm^	0.19 ± 0.01 ^l^	0.26 ± 0.06 ^h^		0.03 ± 0.00 ^g^	3.45 ± 0.01 ^r^	8.35 ± 0.68 ^kl^	0.04 ± 0.04 ^ijk^	0.01 ± 0.01 ^de^	0.02 ± 0.01 ^g^	3.63 ± 0.37 ^ij^	0.05 ± 0.02 ^m^	1.35 ± 0.01 ^b^
	M14	3.81 ± 0.11 ^k^	2.29 ± 0.48 ^jk^	0.02 ± 0.00 ^lm^	0.21 ± 0.03 ^l^	0.30 ± 0.05 ^g^		0.03 ± 0.00 ^g^	3.50 ± 0.66 ^qr^	6.89 ± 0.47 ^lm^	0.04 ± 0.03 ^ijk^	0.01 ± 0.03 ^de^	0.01 ± 0.02 ^h^	6.19 ± 1.13 ^gh^	0.11 ± 0.02 ^hi^	0.71 ± 0.01 ^d^
	M15	4.77 ± 0.13 ^j^	2.02 ± 0.29 ^jkl^	0.03 ± 0.00 ^kl^	0.26 ± 0.01 ^k^	0.33 ± 0.06 ^f^		0.04 ± 0.00 ^f^	4.75 ± 0.97 ^n^	9.97 ± 0.56 ^kl^	0.05 ± 0.02 ^ijk^	0.01 ± 0.02 ^de^	0.01 ± 0.00 ^h^	5.71 ± 0.40 ^h^	0.09 ± 0.00 ^kl^	0.78 ± 0.01 ^c^
	M16	11.16 ± 0.10 ^d^	4.60 ± 0.52 ^f^	0.1 ± 0.00 ^ef^	0.95 ± 0.01 ^e^	0.31 ± 0.1 f^g^		0.07 ± 0.00 ^d^	9.34 ± 0.89 ^j^	21.45 ± 0.59 ^gh^	0.18 ± 0.00 ^de^	0.02 ± 0.02 ^c^	0.04 ± 0.00 ^ef^	12.78 ± 0.91 ^e^	0.08 ± 0.01 ^kl^	0.44 ± 0.02 ^i^
	M17	8.93 ± 0.62 ^f^	8.53 ± 0.43 ^d^	0.1 ± 0.00 ^ef^	0.68 ± 0.01 ^h^	0.50 ± 0.06 ^d^		0.07 ± 0.00 ^d^	8.95 ± 0.69 ^k^	22.56 ± 0.61 ^gh^	0.28 ± 0.01 ^c^	0.03 ± 0.01 ^b^	0.05 ± 0.00 ^d^	16.97 ± 0.44 ^d^	0.2 ± 0.01 ^e^	0.63 ± 0.01 ^e^
	M18	7.04 ± 1.13 ^h^	3.88 ± 0.61 ^g^	0.07 ± 0.00 ^i^	0.88 ± 0.01 ^f^	0.43 ± 0.10 ^e^		0.08 ± 0.00 ^c^	11.00 ± 0.74 ^f^	22.13 ± 1.22 ^gh^	0.17 ± 0.01 ^def^	0.02 ± 0.02 ^c^	0.05 ± 0.01 ^d^	20.91 ± 0.99 ^bc^	0.27 ± 0.01 ^d^	0.53 ± 0.01 ^gh^
	M19	6.37 ± 0.37 ^i^	4.81 ± 0.46 ^f^	0.08 ± 0.00 ^h^	0.91 ± 0.01 ^ef^	0.45 ± 0.07 ^e^		0.08 ± 0.00 ^c^	10.62 ± 0.23 ^h^	33.16 ± 1.17 ^e^	0.20 ± 0.01 ^d^	0.02 ± 0.04 ^c^	0.05 ± 0.00 ^d^	20.96 ± 1.19 ^bc^	0.11 ± 0.01 ^hi^	0.71 ± 0.01 ^d^
	M20	7.16 ± 0.95 ^gh^	5.78 ± 0.93 ^e^	0.09 ± 0.00 ^fg^	0.72 ± 0.01 ^gh^	0.74 ± 0.07 ^b^		0.08 ± 0.00 ^c^	11.40 ± 0.17 ^d^	20.99 ± 0.71 ^h^	0.15 ± 0.01 ^fgh^	0.02 ± 0.07 ^c^	0.04 ± 0.00 ^ef^	22.12 ± 1.22 ^b^	0.09 ± 0 ^jk^	0.78 ± 0.01 ^c^
	M21	7.74 ± 0.18 ^g^	3.74 ± 0.67 ^gh^	0.10 ± 0.00 ^ef^	1.09 ± 0.03 ^d^	0.32 ± 0.07 ^fg^		0.09 ± 0.00 ^b^	12.10 ± 1.27 ^c^	23.09 ± 1.27 ^g^	0.16 ± 0.03 ^efg^	0.02 ± 0.06 ^c^	0.05 ± 0.00 ^d^	19.63 ± 0.73 ^c^	0.07 ± 0.01 ^l^	0.50 ± 0.01 ^h^
	M22	6.27 ± 0.11 ^i^	5.22 ± 0.57 ^ef^	0.07 ± 0.00 ^i^	0.56 ± 0.01 ^i^	0.67 ± 0.01 ^c^		0.08 ± 0.00 ^c^	12.06 ± 1.32 ^c^	16.37 ± 0.87 ^j^	0.18 ± 0.01 ^def^	0.02 ± 0.02 ^c^	0.04 ± 0.01 ^f^	13.93 ± 1.16 ^e^	0.37 ± 0.00 ^b^	0.81 ± 0.01 ^c^
**Compounds**		**Esters (7)**								**Furan (1)**						
		**2-Methylbutanoic Acid Methyl Ester**		**Acetic Acid Ethyl Ester**		**Butanoic Acid Ethyl Ester**		**Ethyl 2-Methylpropionate**		**Ethyl 2-Methylbutanoate Dimer**		**Ethyl Lactate**	**Isovaleric Acid, Methyl Ester**		**2-Pentyl Furan**	
RI		1025.8		910.5		1049.4		979.6		1064.1		1367.1	1038.6		1241.5	
RT		396.1		306.4		422.0		353.5		438.9		914.8	410.0		715.1	
DT		1.2		1.3		1.6		1.6		1.7		1.5	1.2		1.3	
OT (µg/kg)		0.4		5		1		0.1		0.013		3	4.4		5.8	
Odorant Description		Fruity		Fragrant, sweet		Pineapple aroma, fruity		Fruity		Fruity		Caramel, fruity	Fruity		Fruity	
ROAV	M1	36.81 ± 1.15 ^h^		18.05 ± 0.14 ^h^		0.31 ± 0.01 ^l^		9.40 ± 0.21 ^ij^		72.3 ± 2.83 ^c^		0.42 ± 0.01 ^i^	1.85 ± 0.02 ^b^		23.01 ± 0.12 ^ef^	
	M2	39.75 ± 1.06 ^g^		21.97 ± 1.41 ^g^		0.37 ± 0.01 ^l^		14.79 ± 1.01 ^efg^		85.34 ± 4.24 ^c^		0.49 ± 0.02 ^h^	1.51 ± 0.06 ^c^		28.18 ± 1.15 ^b^	
	M3	47.45 ± 0.77 ^e^		36.53 ± 1.70 ^d^		0.40 ± 0.01 ^l^		19.77 ± 1.08 ^c^		91.24 ± 2.83 ^b^		0.53 ± 0.02 ^gh^	1.44 ± 0.04 ^c^		27.40 ± 0.82 ^bc^	
	M4	41.44 ± 0.62 ^fg^		44.4 ± 2.27 ^b^		0.98 ± 0.02 ^ghi^		13.00 ± 1.23 ^gh^		100.00 ± 0.00 ^a^		0.43 ± 0.02 ^i^	1.18 ± 0.10 ^d^		21.52 ± 0.67 ^fg^	
	M5	29.9 ± 0.69 ^i^		30.42 ± 0.89 ^e^		1.04 ± 0.14 ^g^		13.00 ± 1.32 ^gh^		100.00 ± 0.00 ^a^		0.35 ± 0.01 ^j^	0.89 ± 0.10 ^f^		16.81 ± 1.14 ^h^	
	M6	41.44 ± 0.49 ^fg^		51.48 ± 2.03 ^a^		2.28 ± 0.03 ^c^		16.25 ± 1.05 ^de^		100.00 ± 0.00 ^a^		0.43 ± 0.10 ^i^	1.11 ± 0.13 ^de^		19.89 ± 0.85 ^g^	
	M7	14.95 ± 1.14 ^kl^		17.73 ± 0.88 ^h^		2.34 ± 0.08 ^c^		22.10 ± 0.17 ^b^		100.00 ± 0.00 ^a^		0.22 ± 0.02 ^l^	0.33 ± 0.02 ^gh^		7.78 ± 0.99 ^ijk^	
	M8	49.89 ± 1.25 ^d^		25.42 ± 0.81 ^f^		0.53 ± 0.01 ^k^		15.75 ± 0.99 ^def^		80.79 ± 1.41 ^d^		4.38 ± 0.08 ^a^	2.03 ± 0.02 ^a^		36.31 ± 0.85 ^a^	
	M9	16.66 ± 0.76 ^jk^		22.62 ± 0.52 ^g^		2.44 ± 0.06 ^b^		22.75 ± 1.13 ^b^		100.00 ± 0.00 ^a^		0.43 ± 0.02 ^i^	0.33 ± 0.07 ^gh^		9.50 ± 0.14 ^i^	
	M10	5.53 ± 0.02 ^n^		9.33 ± 0.31 ^j^		0.91 ± 0.02 ^i^		9.10 ± 0.14 ^ij^		100.00 ± 0.00 ^a^		0.17 ± 0.06 ^l^	0.15 ± 0.01 ^j^		3.99 ± 0.10 ^l^	
	M11	13.54 ± 0.58 ^l^		16.64 ± 0.90 ^h^		3.36 ± 0.01 ^a^		7.58 ± 0.60 ^j^		100.00 ± 0.00 ^a^		0.18 ± 0.08 ^l^	0.22 ± 0.01 ^ij^		6.00 ± 0.08 ^k^	
	M12	11.14 ± 1.06 ^m^		13.71 ± 0.76 ^i^		1.30 ± 0.03 ^f^		13.93 ± 1.31 ^fg^		100.00 ± 0.00 ^a^		0.28 ± 0.01 ^k^	0.23 ± 0.04 ^hij^		6.20 ± 0.07 ^k^	
	M13	15.66 ± 0.92 ^kl^		13.71 ± 0.99 ^i^		0.95 ± 0.04 ^hi^		9.45 ± 0.75 ^ij^		100.00 ± 0.00 ^a^		0.32 ± 0.01 ^jk^	0.3 ± 0.01 ^ghi^		6.56 ± 0.07 ^k^	
	M14	13.98 ± 1.10 ^l^		18.59 ± 0.57 ^h^		2.08 ± 0.02 ^d^		15.60 ± 0.55 ^def^		100.00 ± 0.00 ^a^		0.35 ± 0.03 ^j^	0.3 ± 0.01 ^ghi^		7.49 ± 0.11 ^jk^	
	M15	18.69 ± 0.71 ^j^		21.61 ± 1.41 ^g^		2.11 ± 0.02 ^d^		13.00 ± 0.18 ^gh^		100.00 ± 0.00 ^a^		0.33 ± 0.01 ^j^	0.37 ± 0.05 ^g^		9.22 ± 0.19 ^ij^	
	M16	41.86 ± 2.51 ^fg^		35.2 ± 1.41 ^d^		0.66 ± 0.09 ^j^		9.85 ± 1.02 ^i^		50.51 ± 1.41 ^g^		0.66 ± 0.01 ^d^	1.12 ± 0.01 ^de^		24.00 ± 0.14 ^de^	
	M17	43.17 ± 1.98 ^f^		35.88 ± 1.41 ^d^		0.70 ± 0.01 ^j^		14.1 ± 0.42 ^fg^		81.33 ± 1.41 ^d^		0.94 ± 0.01 ^b^	1.04 ± 0.01 ^e^		27.10 ± 1.13 ^bc^	
	M18	48.51 ± 1.84 ^de^		39.01 ± 1.13 ^c^		1.02 ± 0.02 ^gh^		17.02 ± 1.08 ^d^		78.56 ± 1.41 ^d^		0.57 ± 0.01 ^fg^	0.85 ± 0.01 ^f^		23.65 ± 0.85 ^e^	
	M19	61.95 ± 1.41 ^b^		34.97 ± 1.13 ^d^		0.71 ± 0.01 ^j^		10.62 ± 0.72 ^i^		54.46 ± 0.64 ^f^		0.59 ± 0.02 ^ef^	0.8 ± 0.01 ^f^		23.99 ± 2.26 ^de^	
	M20	62.96 ± 0.36 ^b^		40.44 ± 0.35 ^c^		0.74 ± 0.01 ^j^		11.11 ± 1.26 ^hi^		85.47 ± 2.12 ^c^		0.74 ± 0.04 ^c^	0.84 ± 0.01 ^f^		24.90 ± 1.28 ^de^	
	M21	73.95 ± 2.12 ^a^		45.23 ± 1.08 ^b^		0.37 ± 0.01 ^l^		11.23 ± 0.85 ^hi^		86.40 ± 2.83 ^c^		0.62 ± 0.01 ^de^	1.19 ± 0.30 ^d^		25.69 ± 0.71 ^cd^	
	M22	59.22 ± 1.^41 c^		44.52 ± 0.67 ^b^		1.74 ± 0.10 ^e^		27.87 ± 1.22 ^a^		80.38 ± 2.83 ^d^		0.93 ± 0.01 ^b^	0.79 ± 0.01 ^f^		28.71 ± 1.13 ^b^	

Note: OT and ROAV are the abbreviations of “odor threshold” and “relative odor activity value”, respectively. RI, RT, and DT are the abbreviations of “retention index”, “retention time”, and “drift time”, respectively. “——” means that the flavor types of the substance were not found. The types of flavor thresholds were mainly obtained from https://mffi.sjtu.edu.cn/database (accessed on 18 March 2022), the literature [44,45,46,47,48,49,50,51], and the book [52]. All data were expressed by mean ± standard deviation. Different superscript lowercase letters in the same row indicate statistical differences between samples at *p* < 0.05 level.

## Data Availability

The original contributions presented in the study are included in the article, further inquiries can be directed to the corresponding author.

## Appendix A

**Table A1:** The grain nutritional quality of HB and the effect of variety(V), environment (E) and V × E interaction on them.

No.	Thousand Seed Weight/g	Volume-Weight/gL^−1^	Hardness/kg	Moisture/%	Ash Content/%	Fat/%	Protein/%	Total Starch/%	Amylose/%	Amylopectin/%	Resistant Starch/%
M1	43.05 ± 0.91 ^cd^	845.43 ± 14.45 ^a^	15.57 ± 0.79 ^bc^	8.45 ± 0.47 ^cdef^	2.01 ± 0.03 ^def^	1.69 ± 0.11 ^hij^	8.46 ± 0.33 ^ij^	45.17 ± 0.19 ^h^	23.59 ± 1.61 ^cd^	21.58 ± 1.42 ^n^	22.75 ± 0.34 ^a^
M2	52.08 ± 1.36 ^a^	764.59 ± 4.72 ^f^	15.81 ± 0.83 ^bc^	8.55 ± 0.46 ^cdef^	1.64 ± 0.08 ^kl^	2.62 ± 0.13 ^b^	8.83 ± 0.04 ^h^	55.11 ± 2.78 ^ef^	22.72 ± 0.77 ^de^	32.39 ± 2.01 ^jkl^	14.12 ± 0.89 ^f^
M3	39.12 ± 1.19 ^e^	752.07 ± 2.31 ^g^	15.31 ± 0.33 ^bcd^	7.98 ± 0.78 ^efg^	1.94 ± 0.08 ^efghi^	2.07 ± 0.18 ^cde^	11.32 ± 0.23 ^b^	55.36 ± 1.62 ^de^	21.32 ± 0.34 ^fg^	34.04 ± 1.96 ^ijk^	11.4 ± 0.46 ^h^
M4	51.16 ± 1.29 ^a^	788.79 ± 2.23 ^e^	15.28 ± 0.41 ^bcd^	7.30 ± 0.59 ^g^	1.8 ± 0.13 ^fghijk^	1.74 ± 0.06 ^ghi^	10.39 ± 0.35 ^cde^	59.34 ± 1.75 ^cd^	22.2 ± 0.33 ^ef^	37.14 ± 2.08 ^fgh^	14.38 ± 0.55 ^f^
M5	39.37 ± 0.63 ^e^	763.43 ± 3.56 ^f^	13.26 ± 0.28 ^efg^	8.49 ± 0.54 ^cdef^	1.74 ± 0.06 ^hijk^	1.82 ± 0.06 ^fgh^	8.25 ± 0.20 ^j^	56.99 ± 0.99 ^cde^	16.58 ± 0.43 ^i^	40.41 ± 0.56 ^de^	20.36 ± 0.67 ^c^
M6	39.57 ± 1.59 ^e^	758.77 ± 2.34 ^fg^	11.85 ± 1.18 ^gh^	8.20 ± 0.86 ^defg^	1.98 ± 0.04 ^efg^	3.06 ± 0.12 ^a^	11.98 ± 0.39 ^a^	44.42 ± 1.44 ^h^	16.01 ± 0.09 ^ij^	28.41 ± 1.35 ^m^	7.18 ± 0.24 ^l^
M7	38.95 ± 1.06 ^e^	725.32 ± 4.67 ^h^	13.86 ± 0.88 ^def^	10.20 ± 0.44 ^ab^	2.21 ± 0.18 ^cd^	1.81 ± 0.08 ^fgh^	8.18 ± 0.20 ^j^	59.53 ± 1.64 ^cd^	25.18 ± 0.22 ^b^	34.35 ± 1.42 ^hijk^	9.91 ± 0.12 ^i^
M8	44.40 ± 0.73 ^c^	784.71 ± 4.82 ^e^	16.67 ± 0.69 ^b^	11.02 ± 0.15 ^a^	3.1 ± 0.12 ^b^	2.19 ± 0.18 ^c^	9.65 ± 0.08 ^g^	59.29 ± 2.61 ^cd^	23.23 ± 0.25 ^cde^	36.06 ± 2.86 ^fghi^	11.42 ± 0.44 ^h^
M9	43.67 ± 2.38 ^c^	782.54 ± 3.78 ^e^	12.51 ± 0.53 ^fgh^	8.82 ± 0.21 ^cde^	1.93 ± 0.08 ^efghi^	2.08 ± 0.21 ^cd^	9.59 ± 0.02 ^g^	55.82 ± 2.35 ^de^	24.16 ± 0.25 ^c^	31.66 ± 2.10 ^kl^	12.1 ± 1.01 ^gh^
M10	47.80 ± 0.91 ^b^	781.07 ± 4.09 ^e^	12.53 ± 0.55 ^fgh^	8.63 ± 1.24 ^cdef^	3.79 ± 0.14 ^a^	1.77 ± 0.13 ^ghi^	10.27 ± 0.08 ^de^	57.18 ± 0.31 ^cde^	26.36 ± 0.88 ^a^	30.82 ± 0.57 ^lm^	7.73 ± 0.75 ^kl^
M11	32.24 ± 2.03 ^g^	787.39 ± 1.90 ^e^	11.51 ± 1.13 ^h^	9.13 ± 0.18 ^cd^	1.76 ± 0.08 ^ghijk^	1.61 ± 0.03 ^ij^	10.08 ± 0.21 ^ef^	52.40 ± 1.73 ^fg^	15.22 ± 0.24 ^j^	37.18 ± 1.49 ^fgh^	18.72 ± 1.07 ^d^
M12	44.21 ± 1.87 ^c^	787.43 ± 2.89 ^e^	13.21 ± 1.26 ^efg^	7.27 ± 0.74 ^g^	2.34 ± 0.33 ^c^	1.8 ± 0.11 ^fghi^	10.33 ± 0.09 ^cde^	51.42 ± 1.55 ^g^	22.91 ± 0.02 ^de^	28.51 ± 1.53 ^m^	8.87 ± 0.14 ^j^
M13	45.27 ± 0.95 ^c^	802.09 ± 5.20 ^d^	21.41 ± 1.54 ^a^	8.97 ± 0.30 ^cde^	1.65 ± 0.04 ^kl^	1.54 ± 0.06 ^j^	10.47 ± 0.15 ^cd^	63.38 ± 2.40 ^a^	15.67 ± 0.04 ^ij^	47.71 ± 2.36 ^a^	19.08 ± 0.32 ^d^
M14	51.86 ± 1.99 ^a^	801.18 ± 4.31 ^d^	14.45 ± 0.50 ^cde^	9.11 ± 0.32 ^cd^	1.95 ± 0.08 ^efgh^	1.92 ± 0.05 ^defg^	9.81 ± 0.14 ^fg^	61.71 ± 1.93 ^ab^	16.71 ± 0.18 ^i^	45.00 ± 1.75 ^abc^	8.54 ± 0.65 ^jk^
M15	41.10 ± 1.21 ^de^	813.70 ± 3.16 ^c^	15.61 ± 0.85 ^bc^	8.88 ± 0.15 ^cde^	1.62 ± 0.13 ^kl^	1.99 ± 0.02 ^def^	9.81 ± 0.10 ^fg^	58.46 ± 1.70 ^cd^	15.17 ± 0.22 ^j^	43.29 ± 1.48 ^c^	21.37 ± 0.53 ^b^
M16	35.23 ± 1.36 ^f^	800.68 ± 11.71 ^d^	15.15 ± 0.37 ^bcd^	9.52 ± 0.56 ^bc^	1.80 ± 0.13 ^fghijk^	1.53 ± 0.04 ^j^	10.15 ± 0.20 ^def^	57.09 ± 1.11 ^cde^	22.27 ± 1.08 ^ef^	34.82 ± 0.03 ^ghij^	17.68 ± 0.74 ^e^
M17	39.49 ± 1.60 ^e^	816.45 ± 3.05 ^bc^	15.73 ± 0.72 ^bc^	8.44 ± 0.41 ^cdef^	1.81 ± 0.06 ^efghijk^	2.22 ± 0.13 ^c^	8.77 ± 0.11 ^hi^	59.89 ± 1.39 ^cd^	15.31 ± 0.38 ^j^	44.58 ± 1.77 ^bc^	18.31 ± 0.53 ^de^
M18	45.05 ± 1.09 ^c^	750.43 ± 4.45 ^g^	15.06 ± 0.16 ^bcd^	9.03 ± 0.43 ^cde^	2.04 ± 0.18 ^de^	1.89 ± 0.04 ^defg^	11.06 ± 0.08 ^b^	57.19 ± 0.87 ^cde^	14.99 ± 0.05 ^j^	42.20 ± 0.92 ^cd^	22.49 ± 0.52 ^a^
M19	39.68 ± 1.81 ^e^	756.11 ± 2.13 ^fg^	11.81 ± 0.96 ^gh^	8.51 ± 0.21 ^cdef^	1.88 ± 0.09 ^efghij^	1.69 ± 0.03 ^hij^	10.65 ± 0.06 ^c^	55.71 ± 0.73 ^de^	18.2 ± 0.31 ^h^	37.52 ± 0.43 ^fg^	12.47 ± 0.55 ^g^
M20	44.89 ± 2.02 ^c^	823.57 ± 1.70 ^b^	15.72 ± 0.89 ^bc^	7.30 ± 0.71 ^g^	1.68 ± 0.06 ^jk^	1.91 ± 0.02 ^defg^	10.18 ± 0.29 ^de^	57.47 ± 1.49 ^cde^	19.12 ± 0.39 ^h^	38.35 ± 1.10 ^ef^	8.92 ± 0.09 ^j^
M21	48.36 ± 0.78 ^b^	842.59 ± 2.72 ^a^	13.90 ± 1.23 ^def^	7.60 ± 0.43 ^fg^	1.47 ± 0.05 ^l^	1.88 ± 0.09 ^efgh^	10.07 ± 0.20 ^ef^	57.32 ± 1.34 ^cde^	21.07 ± 1.20 ^g^	36.25 ± 0.14 ^fghi^	9.02 ± 0.05 ^ij^
M22	35.10 ± 0.34 ^f^	845.43 ± 14.45 ^a^	9.75 ± 0.78 ^i^	8.45 ± 0.58 ^cdef^	1.71 ± 0.06 ^ijk^	2.49 ± 0.06 ^b^	11.72 ± 0.107 ^a^	56.69 ± 2.35 ^cde^	10.35 ± 0.29 ^k^	46.35 ± 2.65 ^ab^	4.14 ± 0.10 ^m^
V	64.79 **	28.5 **	43.49 **	34.16 **	12.8 **	46.68 **	28.75 **	27.16 **	30.87 **	17.99 **	24.61 **
E	17.89 **	49.21 **	40.19 **	40.81 **	49.72 **	24.88 **	24.75 **	50.98 **	52.66 **	62.99 **	46.44 **
V × E	16.52 **	21.91 **	14.89 **	20.68 **	36.85 **	27.46 **	46.07 **	20.49 **	16.27 **	18.39 **	28.84 **

All data were expressed by mean ± standard deviation. Different superscript lowercase letters in the same row indicate statistical differences between samples at *p* < 0.05 level. The results with ** indicate extremely significant (*p* < 0.01), the results of analysis of variance (ANOVA) were expressed as a percentage of the total mean square.
